# fastMETA: a fast and efficient tool for multivariate meta-analysis of GWAS

**DOI:** 10.3389/fgene.2025.1718626

**Published:** 2025-12-19

**Authors:** Georgios A. Manios, Dionysios Kandylas, Athanasios Kylonis, Pantelis G. Bagos, Panagiota I. Kontou

**Affiliations:** 1 Department of Computer Science and Biomedical Informatics, University of Thessaly, Lamia, Greece; 2 Department of Mathematics, University of Thessaly, Lamia, Greece

**Keywords:** meta-analysis, GWAS, multivariate, pleiotropy, multiple traits

## Abstract

Genome-Wide Association Studies (GWAS) have transformed human genetics by identifying thousands of loci associated with complex traits and diseases. Yet, individual GWAS are often underpowered, and traditional meta-analysis methods - though widely used in tools such as METAL, GWAMA, and PLINK- typically analyze one trait at a time. This univariate focus risks overlooking pleiotropy and the correlations among traits that underlie complex genetic architectures. To address this gap, we introduce fastMETA, a novel and computationally efficient framework for multivariate meta-analysis of GWAS summary statistics. fastMETA implements an adaptation of the marginal method of moments (MmoM), avoiding the computational burden of hierarchical multivariate models while retaining statistical rigor. Three estimation strategies are provided: (i) a direct replication of the classical MmoM, (ii) a Pearson correlation–based approach, and (iii) a new method that aggregates correlations across all SNPs to estimate a stable trait correlation matrix. This last approach is particularly suited to large-scale GWAS, where within-study correlations are rarely available. We benchmarked fastMETA against existing multivariate meta-analysis packages (mvmeta in R and Stata, xmeta in R) using both real and synthetic datasets. Across scenarios, fastMETA consistently achieved 15–20× faster runtimes while maintaining high concordance with established methods. Applications included: (a) a bivariate meta-analysis of pediatric musculoskeletal traits, replicating pleiotropic effects at the TOM1L2/SREBF1 locus; (b) a multivariate meta-analysis of inflammatory bowel disease gene-expression data, showing near-identical results to published findings; and (c) a large set of genetic association meta-analyses, demonstrating robustness even when within-study correlations were ignored. By combining speed, robustness, and flexibility, fastMETA enables researchers to efficiently explore pleiotropy and complex trait relationships in modern GWAS. Its open-source Python implementation is available both as a standalone tool and as a web service (https://github.com/pbagos/fastMETA), lowering barriers to adoption. Importantly, fastMETA provides a practical and scalable solution for the next-generation of genomic meta-analyses, supporting deeper insights into the genetic basis of multifactorial diseases.

## 1 Introduction

Genome-Wide Association Studies (GWAS) have revolutionized genetic research by identifying thousands of genomic loci associated with complex traits and diseases (Ziegler et al., 2008; Uffelmann et al., 2021). However, individual GWAS often face limitations in statistical power due to sample size constraints. To overcome this issue, meta-analysis has emerged as a powerful tool, combining results from multiple studies to enhance statistical power and identify genetic variants with smaller effects (Evangelou and Ioannidis, 2013).

Meta-analysis is widely used in various fields of medical research and is particularly suitable for GWAS due to its ability to effectively combine evidence from multiple studies (Forero et al., 2019). The meta-analysis can be performed with various methods (Begum et al., 2012) using either Individual Patient Data (IPD) or summary statistics; while IPD provides extensive analytical flexibility, using summary statistics is logistically easier (Ioannidis et al., 2002), circumvents many restrictions regarding data sharing, and has been shown to be equally efficient (Lin and Zeng, 2010). An intermediate solution emerges when a research group has access to limited-sample individual genotype data and aims to integrate it with summary statistics that are available in the public databases and repositories (Kontou and Bagos, 2024). The basic meta-analysis techniques are easily implemented with statistical packages such as Stata and R and are supported by specialized GWAS meta-analysis tools like METAL (Willer et al., 2010), GWAMA (Mägi and Morris, 2010), and PLINK (Purcell, 2007).

Traditional meta-analyses typically examine one trait at a time, potentially overlooking pleiotropic effects and complex correlations among multiple traits. In genetics, pleiotropy is defined as the phenomenon in which a genetic locus can exert influence on several traits or phenotypes (Tyler et al., 2016). Several statistical methods have been used to detect pleiotropy in complex traits (Hackinger and Zeggini, 2017). Such methods are of biological importance, since a joint analysis could help to identify variants that influence both traits, and variants that influence only one of them. On top of that, methods that account for pleiotropy usually offer additional advantages, since they result in increasing statistical power over single trait methods. Multivariate meta-analysis addresses such issues by simultaneously analyzing multiple correlated traits, enabling global hypothesis testing, reducing the burden of multiple comparisons, and improving statistical power (Jackson et al., 2011; Riley et al., 2017). These methods are available in standard statistical packages like Stata and R, but their applicability in GWAS is limited. One reason is the computational demands of the methods, and another is inefficiency of general-purpose statistical packages to handle very large datasets as the ones appearing in GWAS. This paper contributes a novel, computationally efficient multivariate meta-analysis framework for large-scale GWAS, implemented in the fastMETA software. Unlike previous methods, it avoids burdensome within-study covariance estimation and introduces an adaptation of the marginal method of moments (MmoM) (Chen et al., 2016) that uses trait correlations across SNPs to improve speed and robustness. Moreover, it is important to note that the specialized tools we mentioned earlier like METAL, GWAMA or PLINK do not offer such functionality. Our proposed multivariate meta-analysis framework efficiently handles large-scale datasets, enhances power, and supports the discovery of pleiotropy and complex genetic architectures in modern GWAS.

## 2 Materials and methods

Let there be *k* studies and *N* SNPs under consideration. We denote by *p* the number of traits analyzed simultaneously. We denote by ***y***_*ij*_ the vector of the *p* estimates from the *i*th study, with *i* = 1,2, … ,*k* for the *j*th SNP, *j* = 1, 2, … ,*N*. Thus, we have $y_{ij}={\left(y_{1ij},y_{2ij},\ldots ,y_{pij}\right)}^{T}$ and assume that ***y***_*ij*_ is distributed following a multivariate normal distribution around the true means ***β***_*j*_, under the marginal model:

$$y_{ij}∼MVN\left(\beta_{j},\Sigma_{j}+C_{ij}\right)$$ (1)

Here, we denote by ***C***_*ij*_ the within-studies covariance matrix assumed to be known and by ***Σ***_*j*_ the between-studies covariance matrix which is estimated from the data. Usually, each SNP *j* is treated independently. This is the classic model of multivariate random-effects meta-analysis used in numerous applications (van Houwelingen et al., 2002; Jackson et al., 2011; Mavridis and Salanti, 2013). Given an estimate of ***C***_*ij*_ *+* ***Σ***_*j*_ the estimates of ***β***_*j*_ are obtained by the well-known GLS approach:

$${\hat{\beta }}_{j}={\left\{\sum_{i=1}^{k}{\left(\Sigma_{j}+C_{ij}\right)}^{-1}\right\}}^{-1}\left\{\sum_{i=1}^{k}{\left(\Sigma_{j}+C_{ij}\right)}^{-1}y_{ij}\right\}$$ (2)

with estimated variance-covariance matrix given by:

$$\text{var}\left({\hat{\beta }}_{j}\right)={\left\{\sum_{i=1}^{k}{\left(\Sigma_{j}+C_{ij}\right)}^{-1}\right\}}^{-1}$$ (3)

To estimate the between-studies covariance matrix ***Σ***_*j*_ there are several alternatives, such as Maximum Likelihood (ML), Restricted Maximum Likelihood (REML), or the various non-iterative multivariate methods of moments (MM). Although conceptually straightforward, ML/REML estimation require iterative procedures that sometimes encounter convergence problems and require computation time that may be prohibitive for genomics applications, especially in general-purpose statistical packages that cannot handle efficiently large datasets. The MM provides a solution to this problem, however, still requires the within-study correlations, which are often not reported and are difficult to obtain even on request. The marginal method of moments (MmoM) provides an elegant solution by considering the univariate overall estimates ***β*** ^*u*^_*j*_ arising from the traditional method of random effects meta-analysis, the method of moments proposed by DerSimonian and Laird (DerSimonian and Laird, 1986). In this case, Equation 2 becomes the well-known inverse variance estimate

$$\hat{\beta_{tj}^{u}}=\frac{\sum_{i=1}^{k}{\left(s_{tij}^{2}+\tau_{tj}^{2}\right)}^{-1}y_{tij}}{\sum_{i=1}^{k}{\left(s_{tij}^{2}+\tau_{tj}^{2}\right)}^{-1}}$$ (4)

with:

$$\text{var}\left(\hat{\beta_{tj}^{u}}\right)=\frac{1}{\sum_{i=1}^{k}{\left(s_{tij}^{2}+\tau_{tj}^{2}\right)}^{-1}}$$ (5)

In the original MmoM the covariance between estimates is calculated by:

$$\text{cov}\left(\hat{\beta_{tj}^{u}},\hat{\beta_{t^{\prime }j}^{u}}\right)=\sum_{i=1}^{k}\left(\frac{w_{ti}}{\sum w_{ti}}\frac{w_{t^{\prime }i}}{\sum w_{t^{\prime }i}}\left(y_{tij}-\hat{\beta_{tj}^{u}}\right)\left(y_{t^{\prime }ij}-\hat{\beta_{t^{\prime }j}^{u}}\right)\right)$$ (6)

Afterwards, Equations 5, 6 are used to obtain the full variance-covariance matrix $\text{var}\left({\hat{\beta }}_{j}^{u}\right)$ on which we base the inference regarding global tests. This approach is implemented in the xmeta command in R. It is important to note that the method can directly handle missing outcomes under missing completely at random (MCAR) assumption. Thus, studies reporting only one of the traits can be included in the meta-analysis. Note also that Equation 4 through Equation 6 are calculated for each SNP *j* separately, so there is no need for all SNPs to be reported by all included studies. Simulation studies in the original publication showed that the method provides unbiased estimates, well-estimated standard errors, and confidence intervals with good coverage probability. Furthermore, MmoM is found to maintain high relative efficiency compared to conventional multivariate meta-analysis methods fitted with REML or MM, where the within-study correlations are known. This method, which is identical to that implemented in xmeta, is termed Method 1.

A simpler variant (Method 2) would be to calculate the covariance from the simple (unweighted) Pearson correlation of the estimates participating in the meta-analysis of the *j*th SNP. This would lead to:

$$\text{cov}\left(\hat{\beta_{tj}^{u}},\hat{\beta_{t^{\prime }j}^{u}}\right)={\hat{r}}_{jtt^{\prime }}\sqrt{\text{ var}\left(\hat{\beta_{tj}^{u}}\right)\text{var}\left(\hat{\beta_{t^{\prime }j}^{u}}\right)}$$ (7)

Another option, however, that is more suitable for GWAS or gene-expression meta-analyses, would be to use the information from all the SNPs in the particular study:

$${\hat{r}}_{itt^{\prime }}=\text{corr}\left(y_{tij},y_{t^{\prime }ij}\right)=\frac{\sum_{j=1}^{N}\left(y_{tij}-{\bar{y}}_{ti}\right)\left(y_{t^{\prime }ij}-{\bar{y}}_{t^{\prime }i}\right)}{\sqrt{\sum_{j=1}^{N}{\left(y_{tij}-{\bar{y}}_{ti}\right)}^{2}}\sqrt{\sum_{j=1}^{N}{\left(y_{t^{\prime }ij}-{\bar{y}}_{t^{\prime }i}\right)}^{2}}}$$ (8)

Then, after performing similar analyses for each study, a combined estimate of the correlation can be computed by simply averaging:

$${\hat{r}}_{tt^{\prime }}=\frac{\sum_{i=1}^{k}{\hat{r}}_{itt^{\prime }}}{k}$$ (9)

Thus, Equation 9 can then be used to obtain the covariance of the traits *t* and *t’*, $\text{cov}\left(\hat{\beta_{t}^{u}},\hat{\beta_{t^{\prime }}^{u}}\right)$ , which, contrary to Equation 7 is independent of *j* and hence common to all SNPs. This method is called Method 3. In any case, the univariate estimates of Equation 5 are then used to form a p×p diagonal matrix ***S*** ^*u*^ such that

$$S^{u}=\text{diag}\left(\sqrt{\text{var}\left(\hat{\beta_{1j}^{u}}\right)},\sqrt{\text{var}\left(\hat{\beta_{2j}^{u}}\right)},\ldots ,\sqrt{\text{var}\left(\hat{\beta_{pj}^{u}}\right)}\right)$$ (10)

We then use ***S*** ^*u*^ (Equation 10) and R , the matrix of correlation coefficients of the estimates, obtained either by Method 2 or Method 3, to obtain the variance-covariance matrix of the univariate estimates by $\text{var}\left({\hat{\beta }}_{j}^{u}\right)=S^{u}RS^{u}$ . One important issue here is the fact that LD among nearby SNPs can inflate cross-trait correlation estimates. Thus, we need to perform LD-pruning and retain only SNPs with pairwise LD r-squared <0.2. Another consideration could be regarding SNPs with large effect sizes, since these may represent true associations, and consequently may also inflate the estimate of the correlation among summary statistics. Therefore, one may also need to retain for analysis only SNPs with −1.96 < z < 1.96 (Zhu et al., 2015; Li and Zhu, 2017). For other types of analysis, i.e., gene-expression experiments, only the latter option is meaningful. This functionality is included in the tool, and the user can choose the desired values for these cutoffs. One issue that also needs to be clarified is that the estimation of cross-trait correlation needs to be performed only on SNPs present in all studies. This contrasts with the actual meta-analysis, which, as we stated earlier, can be performed for SNPs appearing in some of the studies.

Having obtained the final estimates, it is now very easy to use the well-known multivariate meta-analysis properties to perform global tests. For instance, one may perform a global test for the joint effects, testing the null hypothesis that ***β***_*j*_^*u*^ is zero with a Wald test (Equation 11):

$$W={{\left({\hat{\beta }}_{j}^{u}\right)}^{T}\left(\text{var}\left({\hat{\beta }}_{j}^{u}\right)\right)}^{-1}{\hat{\beta }}_{j}^{u}={\left({\hat{\beta }}_{j}^{u}\right)}^{T}{\left(S^{u}RS^{u}\right)}^{-1}{\hat{\beta }}_{j}^{u}∼\chi_{p}^{2}$$ (11)

Another common application would be to compare two estimates or calculate a confidence interval for their difference, or any other function (Equation 12). The test for ${\hat{D}=\hat{\beta }}_{1}^{u}-{\hat{\beta }}_{2}^{u}=0$ would be:

$$\frac{\hat{D}}{\sqrt{\text{var}\left(\hat{D}\right)}}=\frac{{\hat{\beta }}_{1}^{u}-{\hat{\beta }}_{2}^{u}}{\sqrt{\text{var}\left({\hat{\beta }}_{1}^{u}-{\hat{\beta }}_{2}^{u}\right)}}=\frac{{\hat{\beta }}_{1}^{u}-{\hat{\beta }}_{2}^{u}}{\sqrt{\text{var}\left({\hat{\beta }}_{1}^{u}\right)+\text{var}\left({\hat{\beta }}_{2}^{u}\right)-2\text{cov}\left({\hat{\beta }}_{1}^{u},{\hat{\beta }}_{2}^{u}\right)}}∼N\left(0,1\right)$$ (12)

**FIGURE 1:**
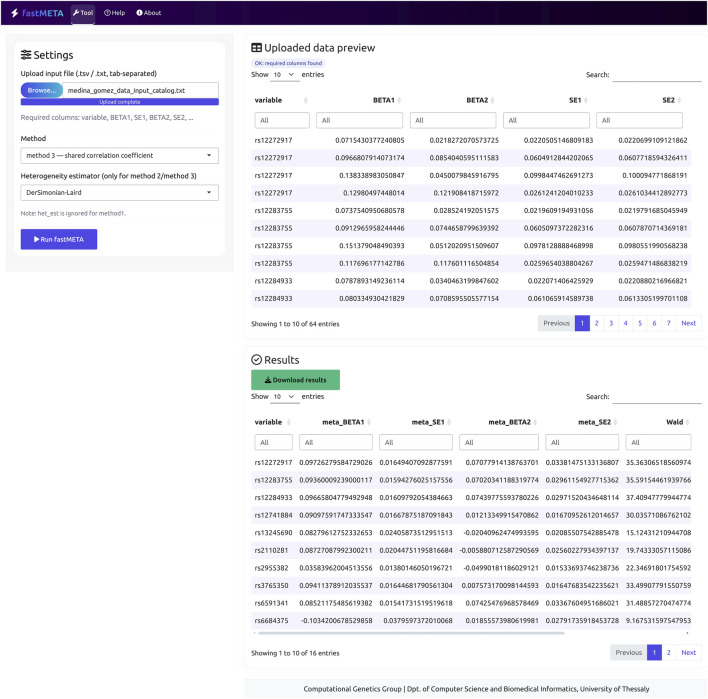
Screenshot from the web interface of fastMETA. In the sidebar panel, the user can select the desired settings to perform a multivariate meta-analysis task and in the main panel, the input file preview and the meta-analysis results, are displayed.

The fastMETA software provides implementations for all three methods, supporting both fixed-effect and random-effects meta-analysis models. We need to emphasize here that although the original MmoM was presented for random effects, and this is apparent in Equations 1–6, the estimate of the between-studies variance does not appear in the calculations so, fixed effects estimates are also possible with all variants of fastMETA, so we include this option in the program. The code is written in Python (version 3.10.6) and is accessible via a public GitHub repository at https://github.com/pbagos/fastMETA. Furthermore, we provide a web version of fastMETA at the following address: https://compgen.dib.uth.gr/fastMETA/, enabling users to apply all methods incorporated within the software (Figure 1). It is important to note that the web version of fastMETA has a limitation of 512 MB input file. This restriction must be considered, to ensure that the meta-analysis process will not be computationally intensive.

## 3 Results

We evaluated the proposed methods using several datasets to demonstrate their performance and practical utility. We first used the bivariate meta-analysis of total-body lean mass (TB-LM) and total-body less head bone mineral density (TBLH-BMD) regions from 10,414 children in 4 studies. This meta-analysis is important because the bivariate method identified pleiotropic effects with variants in the TOM1L2/SREBF1 locus exerting opposing effects on TB-LM and TBLH-BMD (Medina-Gomez, 2017). We also used data from a multivariate meta-analysis of microarrays gene expression datasets in Crohn’s disease (CD) and Ulcerative colitis (UC) (Vennou et al., 2020b), and compared the results against the standard bivariate method used in the original publication (Vennou et al., 2020a). Lastly, we used data from 75 published meta-analyses of genetic association studies published between 2006–2007, which had the full genotype tables available in the publication. In this test set, we used the standard bivariate method for genetic association studies (Bagos, 2008), but we deliberately ignored the within-studies correlations in order to assess the robustness of the method against misspecification.

**FIGURE 2:**
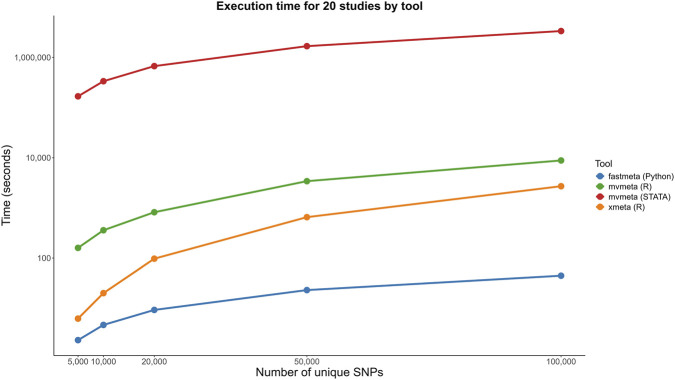
Runtime comparison of fastMETA and other tools for 20 studies and different number of unique variants. In all cases, fastMETA consistently achieves the fastest execution times.

To evaluate computational performance of fastMETA, we generated synthetic datasets with 5, 10, and 20 studies to simulate a large number of unique bivariate meta-analyses (that is, with two traits). Figure 2 compares the runtime of fastMETA against other multivariate meta-analysis packages that use standard methods like REML and MM, including mvmeta (R), mvmeta (STATA), and xmeta (R). While xmeta shows strong performance, fastMETA consistently delivered the fastest execution times, achieving approximately 15–20 times higher speed across all scenarios. It is obvious that the MmoM is more computationally efficient, since xmeta in R is faster compared to mvmeta in the same package (in Stata is even slower). However, a dedicated tool such as fastMETA achieves significant gains, scales better with larger datasets and offers additional functionality. We also evaluated the statistical properties of the method. The MmoM was tested thoroughly in the original publication, both in terms of Type I error rate and power, however method 3 is new and an additional evaluation is warranted, even though the only difference lies in the estimation of the correlation, so we did not expect any deviations. Nevertheless, we simulated a meta-analysis of 5 studies involving two traits and 1,000 SNPs in linkage equilibrium. We sampled z-scores from the bivariate standard normal distribution with correlation equal to 0, 0.25. 0.5 and 0.75 and performed the simulation with 1,000 replications. In all cases the coverage probability ranged from 96.42% to 96.52%, close to the nominal level and in agreement with the original MmoM. All the comparisons were conducted on a personal computer equipped with an Intel i5-10500 processor operating at a base frequency of 3.10 GHz, supported by 16 GB of RAM.

**FIGURE 3:**
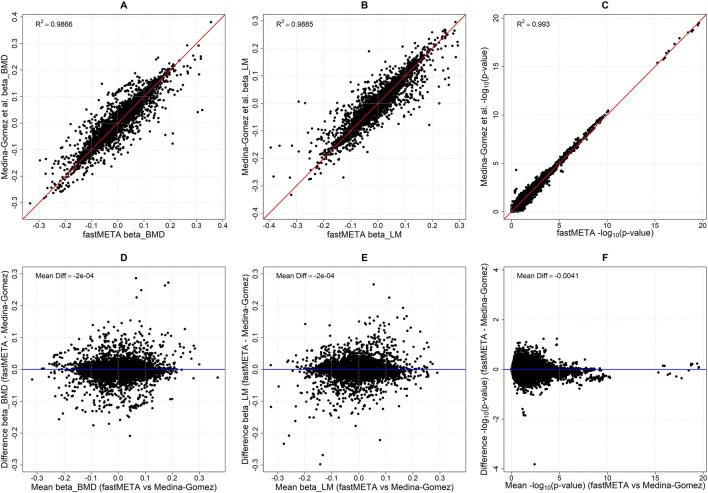
Comparison of **(A,B)** the overall effects and **(C)** the -log_10_ (p-values) from the global test obtained using method 3 of fastMETA combined with LD-pruning, versus those reported in the bivariate meta-analysis results of GWAS Catalog by Medina-Gomez et al. The strong concordance illustrates the high agreement between the two approaches. The second row of panels **(D–F)** shows the respective Bland-Altman plots, where the differences between fastMETA and Medina-Gomez et al. are plotted against their mean, confirming the absence of bias across the full range of the overall effects and -log_10_ (p-values).

For the bivariate meta-analysis of Medina-Gomez et al. we focused on the reported results from the GWAS Catalog database (Sollis et al., 2023). Since in the original publication the fixed-effect meta-analysis was used, we tried to replicate these results using fastMETA, combined with the built-in LD-pruning approach, leveraging LD information from the reference panel of TOP-LD (Huang et al., 2022). As expected, Method 3 achieved the best results, closely matching the published estimates, with no evidence for bias (Figure 3). Similar results were obtained when the z-score cutoff was used alone, or in combination with LD-pruning (data not shown). To further demonstrate the concordance between the two methods, in Table 1 we list the top 20 SNPs with the corresponding p-values obtained with both methods.

**TABLE 1:** Top 20 SNPs from the original Medina-Gomez et al. bivariate meta-analysis, ranked by their reported p-values, shown alongside their corresponding p-values obtained using fastMETA (Method 3, LD-pruning). The table illustrates the concordance between the original meta-analysis results and those derived using fastMETA.

Rank	SNP	p-value (Medina-Gomez et al.)	p-value (fastMETA)
1	rs917727	3.07E-20	2.45E-20
2	rs917726	3.26E-20	2.77E-20
3	rs7776725	5.45E-20	3.14E-20
4	rs3801382	1.72E-19	1.05E-19
5	rs718766	2.23E-19	1.52E-19
6	rs3801387	2.36E-19	1.39E-19
7	rs4727924	3.06E-19	3.32E-19
8	rs2908004	1.19E-18	2.67E-18
9	rs2536189	4.55E-18	8.67E-18
10	rs2536180	2.44E-17	3.56E-17
11	rs2254595	3.88E-17	6.27E-17
12	rs2536182	7.38E-17	1.29E-16
13	rs3779381	1.25E-16	8.90E-17
14	rs2707466	4.06E-16	5.35E-16
15	rs13245690	3.51E-11	7.60E-11
16	rs6950680	4.31E-11	8.33E-11
17	rs7801723	5.15E-11	1.02E-10
18	rs798943	9.86E-11	1.75E-10
19	rs7501812	1.44E-10	2.69E-10
20	rs12706318	1.530E-10	2.81Ε-10

**FIGURE 4:**
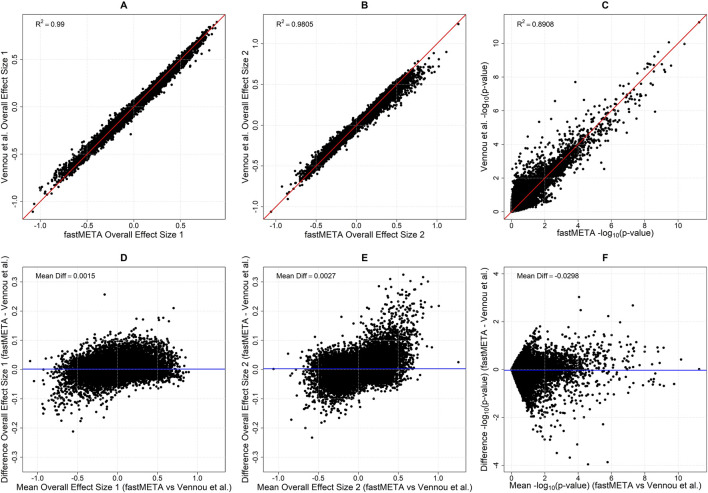
Comparison of **(A,B)** the overall effects and **(C)**–log_10_ (p-values) for the global test obtained using method 3 of fastMETA with a z-cutoff of −1.96 < z < 1.96, versus those reported in Vennou et al. The results show strong agreement between fastMETA and Vennou et al., with nearly identical overall effect sizes (*R*2 = 0.990 and 0.980) and a high correlation in–log_10_ (p-values) (R2 = 0.890), confirming the accuracy of fastMETA. The second row **(D–F)** illustrates the respective Bland-Altman plots, where the differences between fastMETA and Vennou et al. are plotted against their mean. These plots show that the two methods agree consistently, with only minor discrepancies, indicating no bias across the entire range of overall effects and -log_10_ (p-values).

For the microarray meta-analysis in inflammatory bowel disease, we reproduced the random-effects bivariate analysis reported by Vennou et al., using fastMETA with Method 3, applying Hedges’s g correction and the DerSimonian-Laird heterogeneity estimator. In this analysis we applied a z-score cutoff of −1.96 < z < 1.96 for the calculation of cross-trait correlation. The agreement between fastMETA and the original results was very high, as shown in Figure 4. In Table 2, the top 20 genes are presented, with their ranking based on the p-values reported in the Vennou et al. bivariate meta-analysis.

**TABLE 2:** Top 20 genes, from the Vennou et al. bivariate meta-analysis, ranked by their reported p-values, displayed with their corresponding p-values obtained using fastMETA (Method 3, z-cutoff). The table highlights the agreement between the findings of the original study and those produced by fastMETA.

Rank	Gene	p-value (Vennou et al.)	p-value (fastMETA)
1	MAPK14	5.60E-12	5.40E-12
2	GK	8.70E-11	3.51E-10
3	GBP2	1.10E-10	4.12E-11
4	H2BFS	3.00E-10	8.24E-10
5	MUC1	3.20E-10	1.57E-09
6	ALPL	6.00E-10	2.58E-09
7	MMP9	1.60E-09	6.74E-09
8	GNAI3	1.70E-09	9.26E-10
9	KREMEN1	1.70E-09	5.42E-09
10	LIMK2	1.80E-09	4.47E-09
11	USB1	1.90E-09	2.82E-09
12	SOCS3	2.10E-09	2.59E-10
13	OPLAH	2.60E-09	6.84E-09
14	DSC2	3.30E-09	1.01E-08
15	CD55	3.70E-09	6.66E-10
16	XPO6	4.70E-09	2.82E-09
17	SH2D1B	4.90E-09	5.92E-10
18	SLC2A3	6.00E-09	2.72E-08
19	TRIP6	2.00E-08	1.47E-04
20	PGS1	2.40E-08	4.32E-09

**FIGURE 5:**
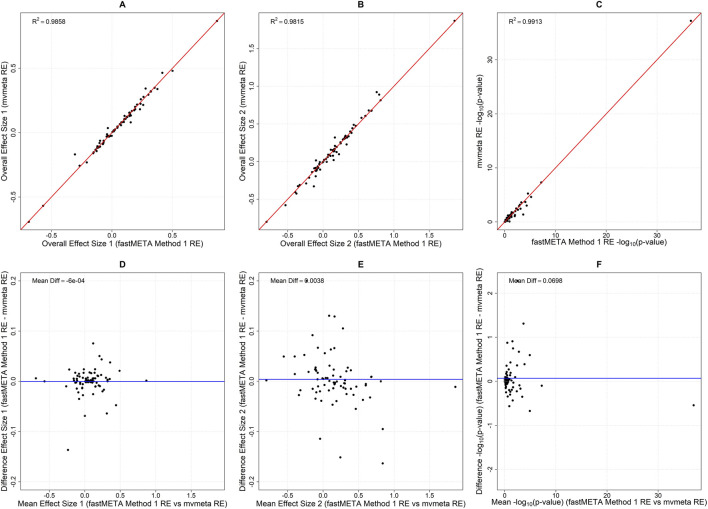
Comparison of **(A,B)** the overall effects and **(C)**–log_10_ (p-values) for the global test obtained using Method 1 of fastMETA with random effects, versus those reported in the 75 studies with mvmeta with random effects. The results show strong agreement between fastMETA and mvmeta, with nearly identical overall effect sizes (*R* ^2^ = 0.985 and 0.981) and a high correlation in–log_10_ (p-values) (*R* ^2^ = 0.991), confirming the accuracy of fastMETA. The second row **(D–F)** illustrates the respective Bland-Altman plots, where the differences between fastMETA and mvmeta are plotted against their mean. These plots show that the two methods agree consistently, with no systematic bias across the entire range of overall effects and -log_10_ (p-values).

Finally, we analyzed the set of 75 published genetic association meta-analyses, applying the bivariate genetic model-free approach. Ignoring within-studies correlations in fastMETA still produced strong agreement with Method 1 (Figure 5) and Method 2 (data not shown), with no evidence of bias, demonstrating the robustness of MmoM. We need to emphasize though, that for this type of analysis, the within studies correlations can be computed analytically from the genotype counts, and hence the MmoM is not optimal; we included this result only for demonstration, and yet the results are compelling. Collectively, these results illustrate that fastMETA achieves both high computational efficiency and high statistical accuracy across diverse datasets, confirming its suitability for large-scale genomic meta-analyses.

## 4 Discussion

We have introduced fastMETA, a novel computational framework for conducting multivariate meta-analysis of GWAS summary statistics. Our tool directly addresses a critical bottleneck in genomic research by providing a method that is both statistically rigorous and computationally efficient. By implementing an adaptation of the marginal method of moments (MmoM), fastMETA bypasses the need for computationally intensive iterative estimation required by hierarchical multivariate models, without sacrificing the ability to detect pleiotropy and account for trait correlations. The core innovation of our approach lies in its flexibility to estimate the trait correlation matrix under different scenarios. While Method 1 provides a direct implementation of the established MmoM, Method 3 is particularly tailored for the genomic context. By aggregating correlation information across all SNPs to derive a stable, genome-wide trait correlation matrix, it offers a powerful solution when within-study correlations are unavailable—a common limitation in practice. Beyond methodological efficiency, our benchmarking confirms that this methodological efficiency translates into substantial practical gains. The consistent 15–20× speed advantage over existing general-purpose tools like mvmeta and xmeta makes large-scale multivariate scans feasible. What was once a prohibitive computation can now be completed in minutes. Of course, specialized tools for GWAS meta-analysis, like METAL, PLINK and GWAMA, may also be equally fast, but they all lack functionality for multivariate meta-analysis.

The proposed method avoids fitting the computationally demanding hierarchical model of Equations 1–3 yet remains robust by replicating its results. fastMETA retains the theoretical properties of the original MmoM, supported by prior simulation studies. We also performed additional simulations using the newly proposed Method 3 that showed similar results in terms of coverage probability. In addition, our extension (Method 3) is well suited to GWAS and other genomic applications, as it uses trait correlation across SNPs, a common and theoretically justified approach in post-GWAS analyses (Dickhaus et al., 2015). We applied fastMETA with Method 3 and replicated two real-world bivariate meta-analyses, one from GWAS and another from microarrays, with remarkable agreement. We also applied Method 1 and 2 in a non-realistic setting (in replicating standard genetic association meta-analysis with a genetic model free method), but even this attempt yielded remarkable agreement with the standard hierarchical models.

Of course, we must have in mind that MmoM is an approximation. Hierarchical multivariate meta-analysis models decompose variability into within- and between-study components, providing richer inference and allowing borrowing of strength across studies (Higgins and Whitehead, 1996). However, these methods often require iterative REML estimation, which can be slow and impractical at GWAS scale. In contrast, the MmoM approach is simpler and faster, and can be easily used even when the within studies correlations are not reported or cannot be calculated. Although there are alternative methods capable of fitting multivariate meta-analyses without the incorporation of within-studies correlation (Riley et al., 2008), these methods are not so efficient and require iterative estimation with the use of Stata or R. An additional advantage is that in typical GWAS settings - where most SNPs are fully observed and the number of studies is small - the benefits of hierarchical models in terms of borrowing strength are minimal. As shown in our applications, simpler methods like MmoM deliver comparable and reliable results with far greater computational efficiency. These findings underscore the practical importance of fastMETA, particularly as genomic datasets continue to expand.

Looking forward, the development of fastMETA aligns with the growing need for scalable bioinformatics tools that can keep pace with the expanding scale and complexity of genomic data. As biobanks and consortium studies continue to grow, meta-analyses will increasingly involve dozens of studies and several correlated traits. The computational efficiency demonstrated by fastMETA is therefore not merely a convenience but a necessity for conducting such large-scale investigations in a practical timeframe. The practical utility of fastMETA is significantly enhanced by its availability as an open-source Python package and a user-friendly web service. The web interface lowers the barrier for adoption for researchers with limited programming experience, allowing a broader community of geneticists and biomedical scientists to apply sophisticated multivariate meta-analysis methods. The 512 MB file size limit on the web server is a reasonable compromise for accessibility and computational resource management, while the standalone version offers unlimited scalability for advanced users with larger datasets.

In conclusion, fastMETA represents a meaningful step towards streamlining multivariate meta-analysis in genomics. By combining statistical rigor with exceptional computational performance and user accessibility, it empowers researchers to more effectively explore the genetic underpinnings of complex traits and diseases. Future developments could include integration with other post-GWAS methods, whereas the tool is quite general requiring minimal input, so it is easy to handle summary statistics from other omics technologies, such as transcriptome - or methylome-wide association studies. For now, fastMETA provides a fast, reliable, and versatile platform that will accelerate discovery of pleiotropic and correlated genetic effects, helping to unravel the complex genetic basis of human health and disease.

## Data Availability

The original contributions presented in the study are included in the article/supplementary material, further inquiries can be directed to the corresponding author.
